# Supplementary material for: The Predictive Value of Radiographs and the Pirani Score for Later Additional Surgery in Ponseti-Treated Idiopathic Clubfeet, an Observational Cohort Study
Source: Children (Basel). 2022 Jun 10;9(6):865. doi: 10.3390/children9060865 (PMC9221593; doi:10.3390/children9060865)
Supplement: Supplementary file 1 [file children-09-00865-s001.zip › children-1737397-supplementary.pdf]

Table S1. Disease-specific instrument for patients with clubfeet.

Item	Response codes
(1) How satisfied are you with the status of your child's foot?	1 = very satisfied, 2 = somewhat satisfied, 3 = somewhat dissatisfied, 4 = very dissatisfied
(2) How satisfied are you with the appearance of your child's foot?	1 = very satisfied, 2 = somewhat satisfied, 3 = somewhat dissatisfied, 4 = very dissatisfied
(3) How often is your child teased because of his or her clubfoot?	1 = never, 2 = sometimes, 3 = usually, 4 = always
(4) How often does your child have problems finding shoes that fit?	1 = never, 2 = sometimes, 3 = usually, 4 = always
(5) How often does your child have problems finding shoes that he or she likes?	1 = never, 2 = sometimes, 3 = usually, 4 = always
(6) Does your child ever complain of pain in his or her [affected] foot?	1 = never, 2 = sometimes, 3 = usually, 4 = always
(7) How limited is your child in his or her ability to walk?	1 = not at all limited, 2 = somewhat limited, 3 = moderately limited, 4 = very limited
(8) How limited is your child in his or her ability to run?	1 = not at all limited, 2 = somewhat limited, 3 = moderately limited, 4 = very limited
(9) How often does your child complain of pain during heavy exercise?	1 = never, 2 = sometimes, 3 = usually, 4 = always
(10) How often does your child complain of pain during moderate exercise?	1 = never, 2 = sometimes, 3 = usually, 4 = always

Table S2. Results according to Richard [9].

excellent result	45 feet (63 %)	
good result	9 feet (13 %)	8 single Achilles tendon lengthenings
		1 three Achilles tendon lengthenings
fair result	18 feet (25 %)	9 tibialis anterior transfers
		2 tibialis anterior transfers with Achilles tendon lengthening and guided growth correction of the anterior distal tibia with an 8-plate
		5 tibialis anterior transfers combined with Achilles tendon lengthening
		1 Achilles tendon lengthening combined with tibia derotation osteotomy
		1 posterior capsule release combined with Achilles tendon lengthening

Table S3. Multivariate regression analysis of all univariate predictors (p > 0.2) of additional surgery.

	B	S.E.	Exp (B)	P
Lat tibia-calcaneal	0.1	0.06	1.1	0.09
Lat talo-calcaneal	0.06	0.06	1.0	0.4
Lat foot dorsiflexion	−0.05	0.05	0.95	0.3
Pirani score before the first cast	0.1	0.37	1.2	0.7
Pirani score before tenotomy	1.1	0.60	3.1	0.06
Pirani score before radiograph	0.1	0.95	1.1	0.9

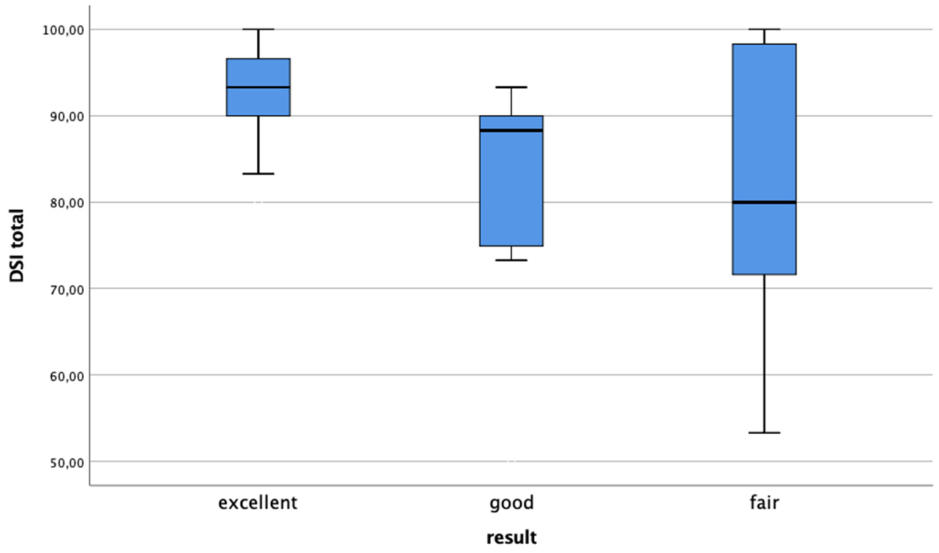

(A) DSI total scores in the different outcome groups (excellent, good, and fair)

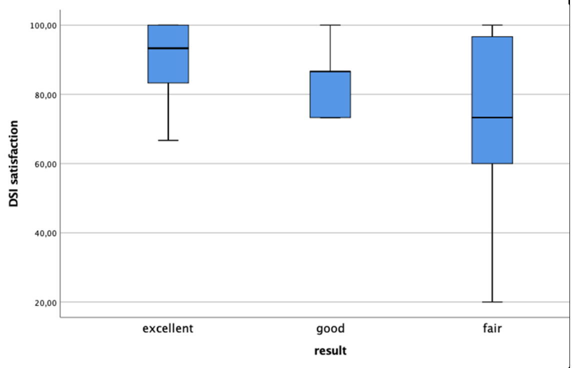

(B) DSI satisfaction scores in the different outcome groups (excellent, good, and fair)

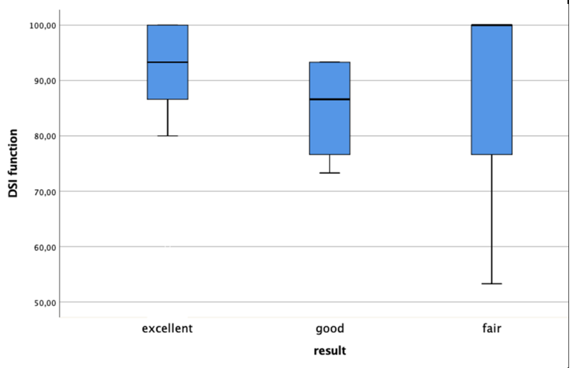

(C) DSI function scores in the different outcome groups (excellent, good, and fair)

Figure S1. Boxplot DSI scores in outcome groups.
